# A Mismanaged Memorial

**Published:** 1895-09-21

**Authors:** 


### Editor's Letter-Box.

[Our correspondents are reminded that proxility is a great bar to publication, and that brevity of style and conciseness of statement greatly facilitate early insertion.]

### A MISMANAGED MEMORIAL.

"A Casual Observer" writes: The result of the Arthur Durham Fund, published in the *British Medical Journal* this week, substantiates the truth of your contention that the Andrew Clark Memorial was mismanaged. The subscriptions for the first-named memorial were raised from amongst old Guy's men, private patients, and friends of Mr. Durham in a few weeks without any demonstration and very little publicity. In no wise has there been any attempt to raise national sympathy through the advocacy of Royal and powerful personages. Yet no less than £1,650 has already been raised out of a sum of £2,000, which it is hoped the subscriptions will reach. The Andrew Clark Memorial has dragged its weary course for nearly three years.

